# Late infectious endocarditis of surgical patch closure of atrial septal defects diagnosed by 18F-fluorodeoxyglucose gated cardiac computed tomography (18F-FDG-PET/CT): a case report

**DOI:** 10.1186/s13104-016-2223-z

**Published:** 2016-08-24

**Authors:** Estelle Honnorat, Piseth Seng, Alberto Riberi, Gilbert Habib, Andreas Stein

**Affiliations:** 1 Assistance Publique - Hôpitaux de Marseille (APHM), Service de Maladies Infectieuses, Hôpital de la Conception, 147, boulevard Baille, Marseille, France; 2 Aix Marseille Univ, INSERM 1095, CNRS 7278, IRD 198, URMITE, Marseille, France; 3 Department of Cardiac Surgery, La Timone Hospital, Aix-Marseille University, 13385 Marseille, France; 4 Department of Cardiology, La Timone Hospital, Aix-Marseille University, Rue Saint-Pierre, 13385 Marseille, France

**Keywords:** Infective endocarditis, Atrial septal defects, Surgical patch closure, *Aggregatibacter aphrophilus*, *Streptococcus intermedius*, PET scan, 18F-FDG-PET/CT, Brain abscess, Diagnosis, Treatment, Infection, Bacteria, Human

## Abstract

**Background:**

In contrast to percutaneous atrial septal occluder device, surgical patch closure of atrial defects was known to be no infective endocarditis risk.

**Case presentation:**

We herein report the first case of late endocarditis of surgical patch closure of atrial septal defects occurred at 47-year after surgery. On September 2014, a 56-year-old immunocompetent French Caucasian man was admitted into the Emergency Department for 3-week history of headache, acute decrease of psychomotor performance and fever at 40 °C. The diagnosis has been evoked during his admission for the management of a brain abscess and confirmed using 18F-fluorodeoxyglucose gated cardiac computed tomography (18F-FDG-PET/CT). Bacterial cultures of surgical deep samples of brain abscess were positive for *Streptococcus intermedius* and *Aggregatibacter aphrophilus* as identified by the matrix-assisted laser desorption/ionization-time of flight (MALDI-TOF) mass spectrometry and confirmed with 16S rRNA gene sequencing. The patient was treated by antibiotics for 8 weeks and surgical patch closure removal.

**Conclusions:**

In summary, late endocarditis on surgical patch and on percutaneous atrial septal occluder device of atrial septal defects is rare. Cardiac imaging by the 18F-fluorodeoxyglucose gated cardiac computed tomography (18F-FDG-PET/CT) could improve the diagnosis and care endocarditis on surgical patch closure of atrial septal defects while transthoracic and transesophageal echocardiography remained difficult to interpret.

## Background

Infectious complication of atrial septal occlude device is rare that represented about 0.1 % of cases [1]. We herein report the first case of endocarditis at 47-year after surgical closure of atrial septal defects discovered by positron emission tomography with 18F-fluorodeoxyglucose gated cardiac computed tomography (18F-FDG-PET/CT).

## Case presentation

**Fig. 1:**
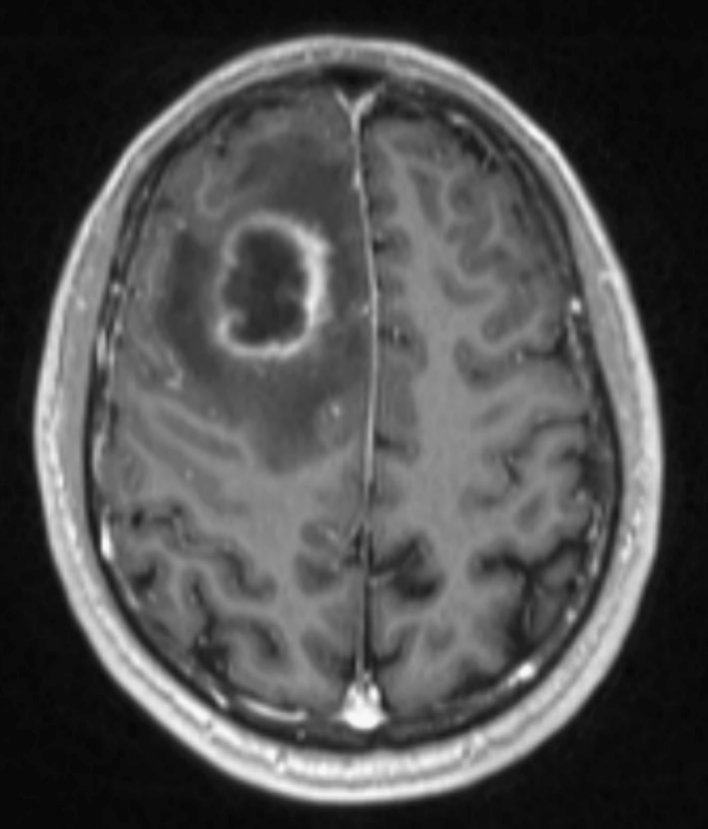
A brain magnetic resonance imaging (MRI) showing a unique brain abscess (27 × 36 × 43 mm)

On September 2014, a 56-year-old immunocompetent French Caucasian man was admitted into the Emergency Department for 3-week history of headache, acute decrease of psychomotor performance and fever at 40 °C. In his medical past-history, he underwent a surgical patch closure of atrial septal defects for patent foramen ovale at 9 years old and dental care at 5 months before his admission. Laboratory investigations revealed an elevated leukocyte count at 12.6 × 10^9^/L with an elevated neutrophil count at 8.4 × 109/L, a normal platelet count at 180 × 10^9^/L, an elevated C reactive protein rate at 97 mg/L and negative blood culture. A brain magnetic resonance imaging (MRI) revealed a right occipital cerebral abscess (27 × 36 × 43 mm, hyper intense T2) (Fig. 1).

He underwent surgical brain abscess drainage. Bacterial cultures of surgical deep samples of brain abscess were positive for *Streptococcus intermedius* and *Aggregatibacter aphrophilus* as identified by the matrix-assisted laser desorption/ionization-time of flight (MALDI-TOF) mass spectrometry and confirmed with molecular identification (16 s gene sequencing). The patient was treated first with an empiric antibiotherapy with cefotaxim 4 g every 8 h associated with metronidazole 500 mg every 8 h and cotrimoxazole 800/160 mg every 8 h. One week later, the antibiotic treatment was modified for ceftriaxone 2 g/day associated with metronidazole 500 mg every 8 h and rifampicin 300 mg every 8 h.

**Fig. 2:**
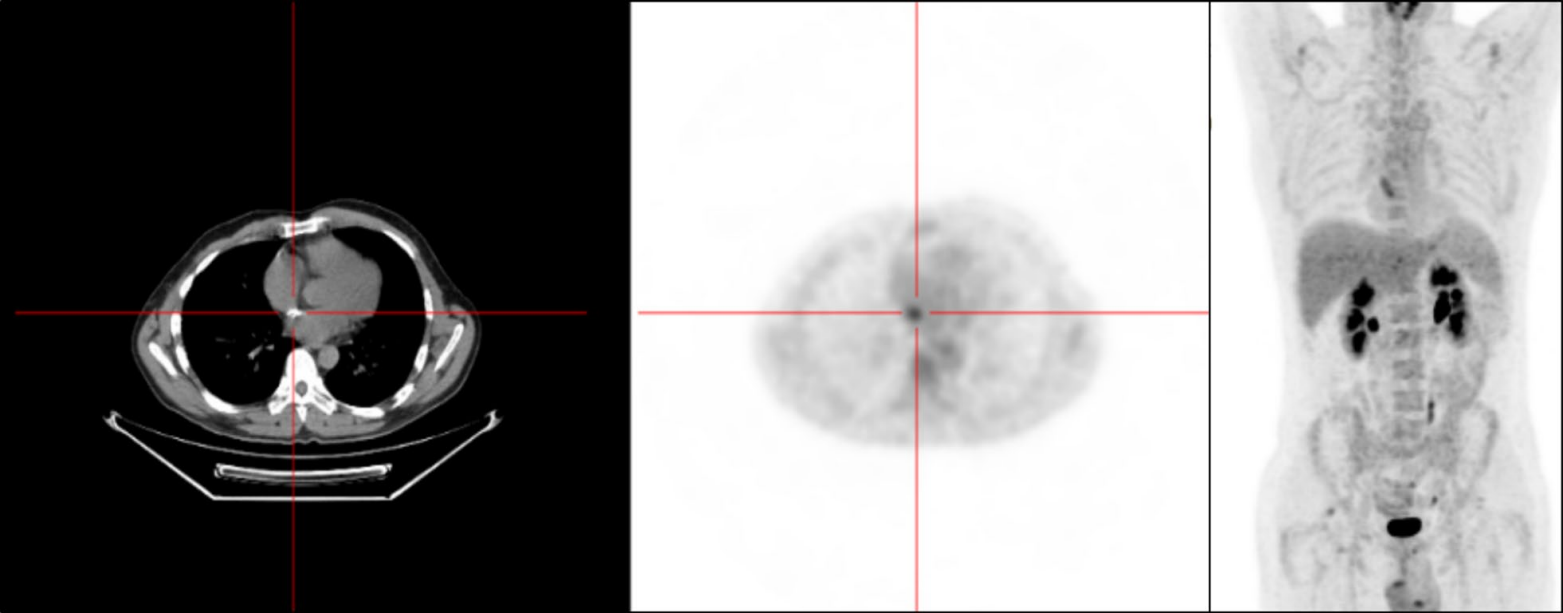
The 18F-fluorodeoxyglucose gated cardiac computed tomography (18F-FDG-PET/CT) imaging showing hypermetabolism hyperactivity around the interauricular septum

A transthoracic echocardiographic examination and a body scan revealed no abnormality. A transesophageal echocardiography showed a high right left shunt suggestive of atrial septal patch dehiscence without direct sign of. An 18F-FDG-PET/CT showed an intense linear hypermetabolism opposite the interauricular septum compatible with an endocarditis on the intracardiac device (Fig. 2). Transesophageal echocardiography after 6 weeks of antibiotic treatment showed a filamentous image opposite the mitral valve and no vegetation image was found. A second 18F-FDG-PET/CT kept showing hypermetabolism on the surgical patch closure of atrial septal defects. The patient was treated by antibiotics for 8 weeks.

At the end of antibiotic treatment transthoracic echocardiographic examination showed an exclusive right left shunt, confirming dehiscence of the surgical patch closure of atrial septal defects. Surgical removal of surgical patch closure of atrial septal defects was necessary on April 2015 with replacement by a bovine pericardial patch. No antibiotics were given during explantation. Explanted atrial surgical patch closure of atrial septal defects looked swollen and calcified with negative bacterial cultures. The patient recovered without further complication. His hemiparesis fully disappeared; he only kept discrete amnesic and attentional failures. He presents a sequelae of attentional and memory deficits at 1 year of follow-up.

## Discussion

In contrast to percutaneous atrial septal occluder device, surgical patch closure of atrial septal defects was known to be associated with no infective endocarditis risk [2]. To our best knowledge, only one case of endocarditis on surgical patch of ventricular septal defect [3] and 17 cases of bacterial endocarditis on percutaneous atrial septal occluder device [4–15] have been reported. In our case, the late onset of the infection occurred at 47-year after surgery, and it suggests that origin of endocarditis of surgical patch closure of atrial septal defects was a hematogenous spread of bacteria during the dental care at 5 months before. Diagnosis of endocarditis on atrial septal occluder device is generally done by transthoracic echocardiographic or transesophageal echocardiography. Therefore, in our case the diagnosis has been evoked during his admission for the management of a brain abscess, and established using 18F-FDG-PET/CT while transthoracic and transesophageal echocardiography remained difficult to interpret. The benefit of 18F-FDG-PET/CT has been demonstrated in the diagnosis of prosthetic valve endocarditis cardiac device-related endocarditis for detect infection by highlighting inflammatory leukocytes express a high density of glucose transporters and are highly metabolically active with the high spatial resolution of cardiac tomography and angiography, and it was recently added to the diagnosis of infective endocarditis criteria [16, 17].

## Conclusion

In summary, late endocarditis on surgical patch and on percutaneous atrial septal occluder device of atrial septal defects is rare. We believe that the contribution of 18F-FDG-PET/CT could improve the diagnosis and care of endocarditis on surgical patch closure or percutaneous atrial septal occluder device of atrial septal defects. A prolonged antibiotherapy and surgical management with surgical patch removal is a major therapeutic option of endocarditis on surgical patch closure of atrial septal defects.

## Abbreviations

MALDI-TOF mass spectrometry: the matrix-assisted laser desorption/ionization-time of flight mass spectrometry
18F-FDG-PET/CT: 18F-fluorodeoxyglucose positron emission tomography/computed tomography
MRI: magnetic resonance imaging
